# Immunohistochemical and Molecular Investigations Show Alteration in the Inflammatory Profile of Multiple System Atrophy Brain

**DOI:** 10.1093/jnen/nly035

**Published:** 2018-04-23

**Authors:** Aoife P Kiely, Christina E Murray, Sandrine C Foti, Bridget C Benson, Robert Courtney, Catherine Strand, Tammaryn Lashley, Janice L Holton

**Affiliations:** Queen Square Brain Bank for Neurological Disorders, Department of Molecular Neuroscience, UCL Institute of Neurology, London, UK

**Keywords:** CX3CL1, CX3CR1, Cytokine, Microglia, Multiple system atrophy, NanoString, Neuroinflammation, Neuropathology

## Abstract

Multiple system atrophy (MSA) is an adult-onset neurodegenerative disease characterized by aggregation of α-synuclein in oligodendrocytes to form glial cytoplasmic inclusions. According to the distribution of neurodegeneration, MSA is subtyped as striatonigral degeneration (SND), olivopontocerebellar atrophy (OPCA), or as combination of these 2 (mixed MSA). In the current study, we aimed to investigate regional microglial populations and gene expression in the 3 different MSA subtypes. Microscopy with microglial marker Iba-1 combined with either proinflammatory marker CD68 or anti-inflammatory marker Arginase-1 was analyzed in control, SND, and OPCA cases (n = 5) using paraffin embedded sections. Western immunoblotting and cytokine array were used to determine protein expression in MSA and control brain regions. Gene expression was investigated using the NanoString nCounter Human Inflammation panel v2 mRNA Expression Assay. Analysis of neuropathological subtypes of MSA demonstrated a significant increase in microglia in the substantia nigra of OPCA cases. There was no difference in the microglial activation state in any region. Cytokine expression in MSA was comparable with controls. Decreased expression of CX3CL1 precursor protein and significantly greater CX3CR1 protein was found in MSA. NanoString analysis revealed the >2-fold greater expression of *ARG1, MASP1, NOX4, PTGDR2*, and *C6* in MSA.

## INTRODUCTION

Multiple system atrophy (MSA) is a sporadic adult-onset progressive neurodegenerative disease characterized clinically by parkinsonism, cerebellar ataxia, and autonomic failure. The disease is confirmed pathologically by accumulated α-synuclein protein within oligodendrocytes forming glial cytoplasmic inclusions as well as variable numbers of neuronal nuclear or cytoplasmic inclusions and regional neurodegeneration. Neuropathological classification of MSA depends on the brain regions most severely affected by neuronal loss: striatonigral degeneration (MSA SND), olivopontocerebellar atrophy (MSA OPCA), or as a mixed form (mixed MSA). Rare neuropathological subtypes are also described, known as minimal change MSA and atypical MSA (1, 2).

Neuroinflammation is a dynamic response, resulting in changes in glial cell function and morphology, upregulated transcription of inflammation linked genes and cytokine production. Neuroinflammation has been identified as a hallmark of neurodegenerative diseases including Alzheimer disease (3, 4), Parkinson disease (5), and dementia with Lewy bodies (6) among others. Proinflammatory neuroinflammation has also been shown to be a normal process of nondiseased aging (reviewed in [7]). We have previously shown that microglia, the key cellular component of the neuroinflammatory response, are increased in number in a group of MSA cases with mixed neuropathological subtypes (8). Others have reported similar upregulation of astrocytes along with microglial activation in MSA brain (9, 10). Microglia had generally been proposed to have a bipolar activation style and in the absence of insult, toxin, or disease, they exist in a ramified state. In their ramified state, microglia have constantly motile processes surveying their local area (11). Microglial processes may then detect different inducers that can stimulate the cells to become activated either to a proinflammatory, phagocytic (M1 state), an anti-inflammatory, prohealing (M2 state), or a “resolution phase macrophage” (rM), which has hybrid expression of both M1 and M2 phenotypic markers (12). In recent years, general consensus has begun to move away from the bipolar theory of microglial activation in favor of a spectrum of activation (13) in which active microglia may fluctuate rapidly through multiple degrees of activation expressing a continuum of variable markers.

Interestingly, a recent study in a mouse model of traumatic brain injury has shown gradual upregulation of M1 marker RNA expression postinjury, while M2 marker expression peaked rapidly postinjury before declining in expression (14). Although this study examined the influence of an acute insult on the dynamics of the M1 to M2 microglial ratio, it might be anticipated that in neurodegeneration an acute response will give way to either a long-term chronic or dysfunctional response. Indeed, both nitrated and aggregated forms of α-synuclein protein, which are present in MSA (15, 16), have been shown to stimulate a pro-inflammatory microglial phenotype (17) and may maintain this activation during the disease course of MSA. We hypothesize that microglia in MSA have become aberrantly activated, potentially exacerbating the development of pathological changes.

We aimed first to investigate whether differences in microglial number, which we previously reported in MSA brain in a group of cases unselected for neuropathological subtype, would be reproduced when analyzing MSA cases by disease subtype. For this, we selected MSA-SND and MSA-OPCA as the 2 disease subtypes showing the widest differential in pathological involvement of different brain regions. We chose substantia nigra because it is severely involved in MSA-SND and cerebellar white matter as a region with extensive pathology in MSA-OPCA. We also included the posterior frontal lobe because this region shows a moderate degree of α-synuclein pathology in both subtypes of MSA. Next, we wished to investigate the activation state of these microglia, whether they be in a pro- or anti-inflammatory state.

To investigate additional aspects of the inflammatory response in MSA, we selected cases of mixed MSA to minimize any influence of brain regional variability in pathology. The functional output of the inflammatory response can be quantified and assessed by assaying the expression of cytokines and chemokines. To understand whether the functional inflammatory output differs between control and MSA, we compared the expression of 36 cytokines and chemokines using commercially available assay systems. CX3CL1 or fractalkine is a chemokine which, together with its specific receptor CX3CR1, is abundantly expressed in the CNS, mediating neuron-microglial interaction (18). The glycosylated form of the neuronal CX3CL1 can bind microglial CX3CR1 and prevent the activation of potentially harmful inflammatory microglial phenotypes (19). The expression of CX3CL1 and CX3CR1 can be altered in the presence of injury or insult (20–22) and impaired CX3CR1 function may exacerbate neurodegeneration (23). To investigate whether similar changes contribute to the inflammatory process in MSA, we determined the expression of CX3CL1 and CX3CR1 in brain tissue. Finally, to provide a wider understanding of the inflammatory response in MSA, we examined the expression of 249 genes associated with inflammation in disease cases compared with controls using NanoString nCounter technology (24). In this way, we aimed to gain a broad understanding of the neuroinflammatory characteristics of MSA to gain insights into disease mechanisms and provide information contributing to the development of disease biomarkers and therapeutic targets.

## MATERIALS AND METHODS

### Case Selection

Cases were selected for each part of the study (immunofluorescence, cytokine arrays, Western immunoblotting, and NanoString analysis) based on diagnosis, tissue availability, gender and age pairing and postmortem delay, we sought to exclude cases that had concomitant pathologies that might exacerbate any inflammatory response. Due to these restrictions, we were unable to use exactly the same cohort for the entire investigation (Supplementary Data Tables S1 and S2).

### Human Brain Tissue

We used brain tissue from cases donated to the Queen Square Brain Bank for Neurological Disorders, UCL Institute of Neurology. The brain donation program and protocols have received ethical approval for research by the NRES Committee London—Central and tissue is stored for research under a license issued by the Human Tissue Authority (No. 12198).

According to routine protocols, one-half of the fresh brain was sliced in the coronal plane, flash frozen, and stored at −80°C. The remaining half brain was fixed in 10% buffered formalin before being sliced in the coronal plane. Tissue blocks were selected for paraffin wax embedding and histology. This material was used for multi-immunofluorescence studies. Frozen tissue was dissected from the posterior frontal cortex and the cerebellar white matter and either homogenized for Western immunoblotting or lysed and processed for total RNA using the RNeasy mini prep kit according to the manufacturer’s instructions (Qiagen, Manchester, UK).

Pathological confirmation of MSA subtype diagnosis based on the Ozawa criteria (25) was performed using paraffin embedded sections (8 μm) stained for relevant markers (including hematoxylin and eosin and Luxol fast blue/cresyl violet and α-synuclein). Multi-immunofluorescence was performed using isotype specific antirabbit IgG, antimouse IgG, or antigoat IgG secondary antibodies conjugated with either Alexa 488 or 594 fluorescent dyes (1:400) (Life Technologies, Paisley, UK) followed by quenching of autofluorescence with 0.1% Sudan black/70% ethanol (Sigma-Aldrich, Dorset, UK) solution for 10 minutes and mounting with glass coverslips using VECTAshield mounting media with 4’,6-diamidino-2-phenylindole (DAPI) nuclear stain (Vector Laboratories, Peterborough, UK). Images were visualized using fluorescence microscopy (Leica DM5500 B).

### Microglial Cell Counting

We selected age matched control, SND, and OPCA cases (n = 5 each) for analysis. For each case, we chose severely affected brain regions: subcortical white matter of the frontal lobe, substantia nigra, and cerebellar white matter. Sections (8 μm) were probed with Iba-1, CD68, and arginase-1. In each region, 5 areas were randomly selected according to a predesigned zig-zag pattern and we counted the number of cells that stained positively for Iba-1 as a marker of total microglia and were of a suitable size and morphology, present in the field of view at 20× magnification. Area of field of view at 20× using an eyepiece magnification of 25× was calculated to be 1.19 mm^2^ and all cell counts were expressed as number of cells counted/mm^2^. In addition, we assessed the number of cells per field at 20× that showed colocalization by multi-immunofluorescence microscopy of either Iba-1 and CD68 as a marker of pro-inflammatory microglia or Iba-1 and Arg-1 as a marker of anti-inflammatory microglia.

### Human Cytokine Array

To analyze cytokine expression in control (n = 6) and mixed MSA (n = 6) we selected posterior frontal cortex as a region showing pathological involvement and used the human cytokine array A (R&D Systems, Abingdon, UK) according to the manufacturer’s instructions. Briefly, nitrocellulose membrane spotted in duplicate with 36 cytokine and chemokine capture antibodies was washed and blocked in blocking buffer for 1 hour at room temperature. For each case, 300 μg of frontal cortex sample was used combined with blocking buffer and human cytokine array A detection antibody cocktail. The membrane was incubated in the sample mixture overnight at 4°C. The membrane was then washed and incubated with Odyssey fluorescent conjugated secondary antibody (IRDye 800CW Streptavidin 926-32230) for 30 minutes at room temperature and visualized by LiCOR Odyssey.

### Western Immunoblotting

Frozen tissue was dissected from control (n = 7) and mixed MSA (n = 7) posterior frontal lobe and homogenized in a high salt buffer pH 7.4 (50 mM Tris–HCL, 175 mM NaCl, 1% Triton-X with protease, and phosphatase inhibitors [Roche, Burgess Hill, UK]) using the Precellys 24 with CKmix ceramic beads (Bertin Technologies, Paris, France). The resulting homogenate was then spun at 1000*g* for 10 minutes at 4°C, the supernatant of which was aliquoted, and protein concentration was determined by BCA assay (Pierce, Thermo Fischer Scientific, Rockford, IL). Samples were run at 120 V through a 7%–12% Bis–Tris gel using the XCell SureLock Mini-Cell Electrophoresis system (ThermoFisher Scientific) and transferred onto nitrocellulose membrane (GE). Membranes were blocked for 1 hour at room temperature using Odyssey blocking buffer (LI-COR, Lincoln, NE) and probed overnight at 4°C with either CX3CL1, CX3CR1 (Proteintech, Manchester, UK), Iba-1 (Wako, Japan), or βIII-tubulin antibodies. Membranes were then washed 3 times in 1× phosphate buffered saline solution containing 0.1% Tween and probed with the appropriate fluorescent conjugated secondary antibody (LI-COR, US0041) for 1 hour at room temperature. Membranes were washed 3 times in 1× phosphate buffered saline solution without tween and results were visualized using Odyssey imaging system (LI-COR).

### RNA Isolation

Total RNA was isolated from posterior frontal lobe and cerebellar white matter homogenates using RNAeasy mRNA kit (Qiagen) according to the manufacturer’s instructions. RNA quantity and quality was assessed using a BioSpectrometer (Eppendorf, Stevenage, UK).

### NanoString nCounter Analysis

To examine the inflammatory environment of MSA mRNA expression was determined using the NanoString nCounter Human Inflammation panel v2 mRNA Expression Assay (NanoString Technologies). Total mRNA was isolated from control (n = 6) and mixed MSA (n = 6) posterior frontal lobe and cerebellar white matter (Supplementary Data Table S2) The protocol was carried out by evaluation project technicians at the Seattle NanoString facility as a proof of concept study using 150 ng of total RNA from each sample following their commercial protocol. Data were collected using the nCounter Digital Analyser.

### Data Analysis

Microglial cell and activated microglial cell counts of all 5 fields of view were taken for each region and each case, GraphPad Prism software was used to perform nonparametric Kruskal-Wallis ANOVA with Dunn’s multiple comparison post hoc test to compare control and disease cases and determine whether any changes between groups reached significant difference when p value was adjusted for multiple comparison to an alpha of 0.05. Intrarater reliability of all microglial counts was assessed using an intraclass correlation coefficient (ICC). A minimum of 10% of cases was recounted by the rater and the ICC was calculated as 0.98, which indicates excellent repeatability. ICC was calculated using a free online calculator http://www.danielsoper.com/statcalc/calculator.aspx?id=42.

Human cytokine array data were analyzed using Image J plug in protein array analyzer which was used according to the developer’s instructions (http://image.bio.methods.free.fr/ImageJ/?Protein-A.). Densitometry of Western immunoblotting results was performed using Image J gel analyzer. Normalized densitometry results for each of the 36 cytokines and chemokines were analyzed using multiple *t* tests using the 2-stage linear set-up procedure of Benjamini, Krieger, and Yekutieli, with Q=1%. Each row was analysed individually, without assuming a consistent standard deviation in order to determine any significant difference between MSA and control.

All analysis of NanoString data was performed using NanoString nSolver software. Data obtained from NanoString nCounter expression counts were inspected and any samples determined to be outliers relative to negative controls were excluded from further analysis. According to the manufacturer’s instructions, the count data were normalized to negative controls and to positive controls that corrected for differences in hybridization efficiency and processing variables, including purification and RNA/reporter complex immobilization. In addition, normalization of mRNA content was performed using housekeeping genes (GAPDH, GUSB, HPRT1, PGK1, TUBB). Two-tailed Student *t* tests were then used to test for a significant difference between gene expression in MSA compared with control posterior frontal lobe and cerebellar white matter. In order to correct for false discovery rate, the Benjamini-Yekutieli method was used to exclude any false positive results.

## RESULTS

### Microglia in MSA

**FIGURE 1.**
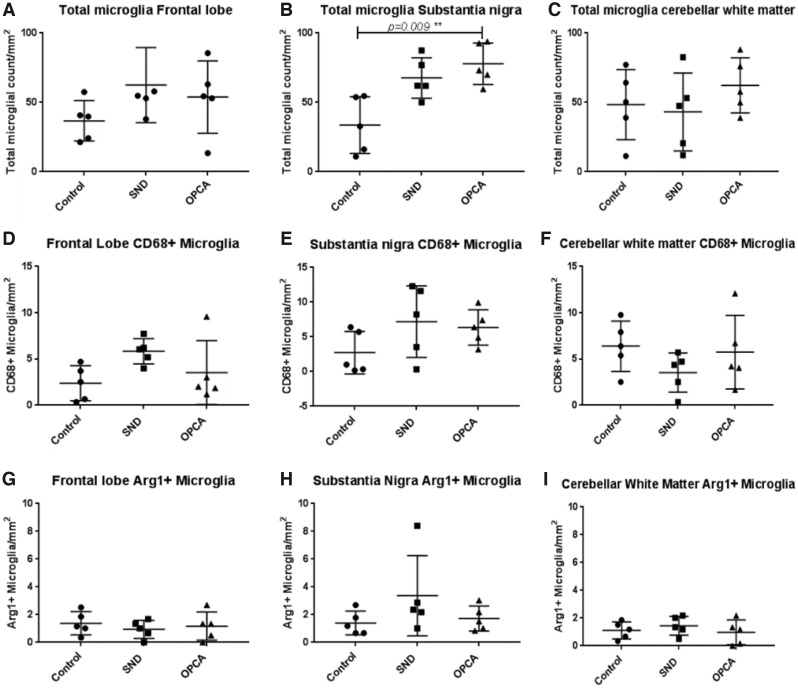
Quantification of microglia in MSA. Analysis of microglial (Iba-1+) cells, activated (CD68+/Arginase-1+), proinflammatory (CD68+) and anti-inflammatory (Arginase-1+) microglia in control and MSA frontal lobe **(A, D, G),** substantia nigra **(B, E, H)** and cerebellar white matter **(C, F, I)**. No significant difference was found in SND and OPCA frontal lobe or cerebellum compared with control or between disease subtypes **(A, C)**. A significantly greater number of microglia were detected in the substantia nigra of OPCA cases compared with control **(B)**. The number of proinflammatory microglia (CD68+) showed no difference in frontal lobe **(D)** substantia nigra **(E)** or cerebellar white matter **(F)** compared with control. No significant difference was found in SND or OPCA in the number of anti-inflammatory (Arginase-1+) microglia in frontal lobe **(G)**, substantia nigra **(H),** or cerebellar white matter **(I)** compared with control using Kruskal-Wallis nonparametric tests with Dunn’s post hoc test for multiple comparisons.

For each brain region the number of Iba-1-positive microglia in each case in controls, MSA-SND, and MSA-OPCA was counted. Details of cases used for this part of the study can be found in Supplementary Data Table S1. Representative field of view images of Iba-1 immunoreactivity with either CD68 or arginase-1 immunoreactivity can be seen in Supplementary Data Figure S1, arrows indicate cells which were counted as double immunoreactive. We demonstrated a significant increase in total microglial number in the substantia nigra of OPCA cases (p = 0.009), the number of microglia in SND substantia nigra approached a significant increase in MSA but the p value lacked significance when adjusted for multiple comparisons (p = 0.08). There was no significant difference in the number of microglia in MSA frontal lobe (SND p = 0.26, OPCA p = 0.43) or cerebellar white matter compared with control (SND p = 0.99, OPCA p = 0.87) when the p values were adjusted for multiple comparisons (Fig. 1A-C). In order to determine whether pro- or anti-inflammatory microglia were differentially distributed in brain regions in MSA subtypes and controls the number of cells coexpressing Iba-1 and CD68 (proinflammatory) and those coexpressing Iba-1 and Arginase-1 (anti-inflammatory) was assessed. Activated microglia with a proinflammatory and anti-inflammatory profile were identified in controls and both MSA subtypes in all brain regions examined. No significant differences were found in the number of pro- or anti-inflammatory microglia in any of the brain regions or MSA subtype when compared with controls (Fig. 1D–I). Differences between groups were analyzed using Kruskal-Wallis ANOVA with Dunn’s post hoc test.

### Chemokine and Cytokine Expression in MSA

**FIGURE 2.**
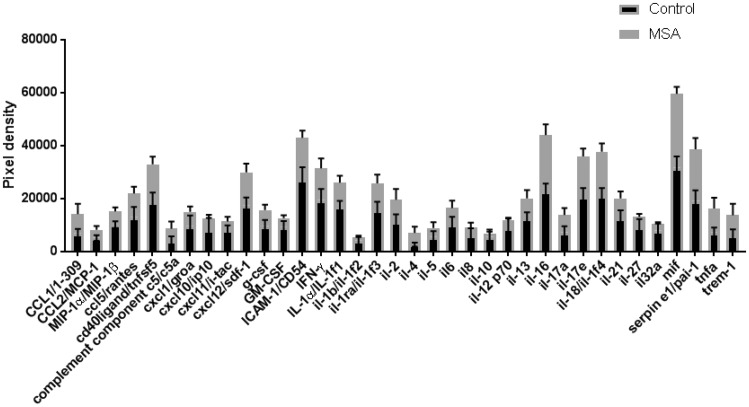
Cytokine expression. Statistical analysis of cytokine array data from a panel of 36 cytokines by multiple *t* tests using the 2-stage linear set-up procedure of Benjamini, Krieger, and Yekutieli, with Q=1%, showed no significant alteration in cytokine expression in MSA compared with control.

Next, we aimed to understand how cytokine and chemokine expression, as a measure of inflammatory activity, might be altered in MSA. Using a human cytokine array, we analyzed the expression of 36 cytokine and chemokine targets of interest in MSA (n = 6) compared with control (n = 6). To minimize any influence of MSA subtype, we chose mixed MSA cases and investigated frontal lobe tissue (Supplementary Data Table S2), using multiple *t* tests using the 2-stage linear step-up procedure of Benjamini, Krieger, and Yekutieli, with Q = 1%. Each row was analyzed individually, without assuming a consistent standard deviation. The array showed no significant alteration in the expression of cytokines and chemokines analyzed between control and MSA cases (Fig. 2).

### CX3CR1 and CX3CL1 Expression in MSA Compared with Control Brain

**FIGURE 3.**
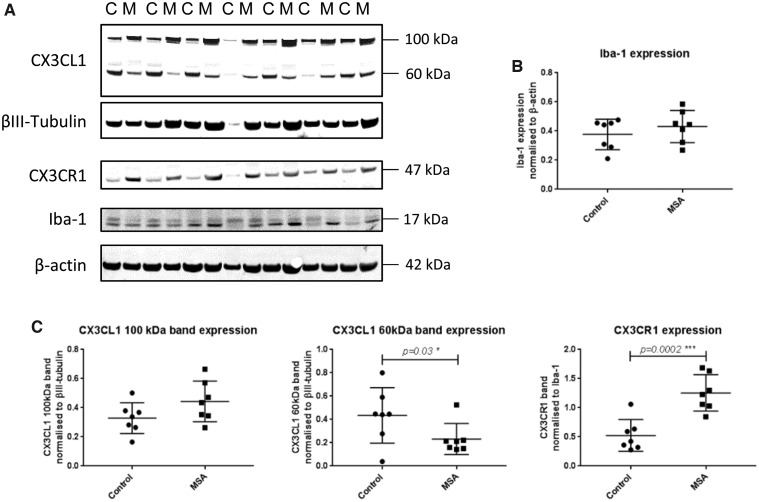
Quantification of Iba-1, CX3CL1, and CX3CR1 protein expression in MSA. **(A)** Representative Western immunoblots in control [C] and MSA [M] frontal cortex. **(B)** No significant alteration was detected in the expression of Iba-1 in MSA compared with control. CX3CL1 was detected at the predicted molecular weight of ∼100 kDa **(A)**, which showed a trend toward upregulation without significance **(C)**. The lower molecular weight band representing the soluble form of the protein was detected at 60 kDa **(A)** and was significantly decreased in MSA when normalized to βIII-tubulin (**C,** p = 0.03). CX3CR1 was detected at the predicted molecular weight of 47 kDa **(A)** and showed a highly significant increase in expression in MSA brain compared with control when normalized to Iba-1, determined by Student *t* test of MSA compared with control (**C**, p = 0.0002).

Using Western immunoblotting, we compared the expression of CX3CL1 and CX3CR1 in mixed MSA (n = 6) and control (n = 6) frontal lobe (Supplementary Data Table S2 and Fig. 3). To control for neuronal and microglial tissue content, we normalized CX3CL1 (expressed by neurons) to the neuronal marker βIII-tubulin and CX3CR1 (expressed by microglia) to the microglial marker Iba-1. We did not observe a significant difference in the expression levels of Iba-1 in MSA compared with control when normalized to β-actin (Fig. 3A, B). At the predicted molecular weight of 90–100 kDa, membrane-bound, glycosylated CX3CL1 expression was not significantly altered in MSA, while an additional band representing the soluble nonglycosylated form of the protein detected at 60 kDa was found to be significantly decreased in MSA (Fig. 3C, p = 0.03). Expression of CX3CR1 showed a highly significant increase in expression in MSA compared with control brain analyzed by Student *t* test (Fig. 3C, p = 0.0002).

### Inflammatory Gene Expression Determined Using NanoString

To characterize further the inflammatory environment in mixed MSA brain compared with controls, we used NanoString nCounter technology to determine the expression of 249 human inflammation associated mRNAs. Supplementary Data Figure S2 shows a heat map diagram using hierarchical clustering of differential gene expression in control and MSA frontal lobe (A) and cerebellar white matter (B). Of the total 249 genes detected by the panel, 9 genes showed significant alteration in mRNA expression in MSA frontal lobe compared with control (Supplementary Data Table S3). Of these, 5 genes were found to have a fold change of expression >2 when compared with control. In the cerebellar white matter 1 gene was found to have a fold change of expression >2 (Table). We chose to use a 2-fold change in either upregulation or downregulation of a gene as the cut-off point to identify genes of interest. By reporting both the significance in terms of p value and the fold change in gene expression, we intended to represent both the statistical significance and variability in terms of p value and biological significance of the change which is more accurately represented by fold change (26).We interpret that the altered expression of *ARG1, MASP1, NOX4, PTGDR2*, and *C6* is likely to be relevant compared with control.

**TABLE.** Frontal Lobe mRNA Expression Determined to be Significantly Altered with >2-Fold Change in MSA Compared with Control

Gene Name	Upregulated in MSA	Downregulated in MSA	Significance (p value)	Fold Change	Protein Encoded and Function
Frontal lobe					
*ARG1*	–	Y	0.0215	3.6	Arginase-1: Anti-inflammatory (57)
*MASP1*	Y	–	0.0437	2.64	Mannan-binding lectin serine protease 1; Proinflammatory (58)
*NOX4*	Y	–	0.0472	2.28	NADPH oxidase 4: Proinflammatory (54)
*PTGDR2*	–	Y	0.0186	2.1	Prostaglandin D2 Receptor 2: Proinflammatory (59)
Cerebellar white matter					
*C6*	Y	–	0.0436	2.88	Complement component 6: Proinflammatory

## DISCUSSION

We have shown that microglia, the key signaling cells in the neuroinflammatory response, are of significantly greater number in the substantia nigra of OPCA cases. Microglial activation to a pro- or anti-inflammatory phenotype was not increased above controls. Consistent with these results, we did not observe significant alteration in the expression of microglial marker Iba-1 protein or in the expression of cytokines and chemokines in MSA compared with control. Investigating the CX3CL1/CX3CR1 neuronal pathway of microglial regulation, we found that CX3CR1 was increased in MSA indicating a neuron-mediated anti-inflammatory response. By analyzing expression of genes associated with inflammation, we confirmed that *ARG1, MASP1, NOX4, PTGDR2*, and *C6* were significantly altered in MSA. These findings indicate subtle modulation of the regulation and expression of inflammatory activity in MSA but these functional changes may not always be reflected by analysis of microglial populations.

It has previously been documented that microglia are increased in MSA (8, 27). Our previous study using a case cohort, which included MSA-mixed, MSA-SND, and MSA-OPCA cases, showed a significantly larger number of microglia in MSA cerebellar and frontal white matter compared with control (8). In this study, we investigated MSA-OPCA and MSA-SND cases separately to determine whether regional microglial burden and activation is altered in these 2 disease subtypes that display the most marked regional differences in pathological changes. We chose substantia nigra (severely affected in MSA-SND), cerebellar white matter (severely affected in MSA-OPCA), and frontal lobe, which is less severely affected in both subtypes. The results of the current study show a significantly greater number of microglia in the substantia nigra of OPCA cases but no significant increase in other brain regions or in SND cases after stringent statistical correction for multiple comparisons. As our previous study pooled together the MSA subtypes, this indicates that caution should be exercised when analyzing regional pathological features in MSA. We did not observe any significant alteration in the expression of Iba-1 protein, as a marker of microglia, in MSA. Although we only investigated Iba-1 protein expression in the frontal lobe, this data further corroborate our evidence that there is no significant increase in microglial number in the majority of brain regions analyzed in MSA. Methodological differences may also contribute to variability between studies. In the current study, we employed a multi-immunofluorescence method as we also wished to determine the activation status of the microglia, unamplified immunofluorescence is a less sensitive method than DAB based immunohistochemistry (28) and so these results are not directly comparable with the previous data. The substantia nigra showed the largest population of microglia in OPCA cases, this is consistent with the work of others who have shown microglial upregulation to be variable and region specific (27). A key aim of this study was to determine whether microglia activated to have pro- or anti-inflammatory functions are altered in MSA. We found no differences in the numbers of microglia in each of these activation states when compared with controls. This supports our finding that there is little alteration in total microglial numbers as microglia have generally been shown to proliferate when they become activated (29). Our findings suggest that microglial proliferation and activation might not be a key component of the inflammatory response in MSA. This is consistent with recent work that used the MSA mouse model to inhibit microglial myeloperoxidase, although this resulted in less microglial activation it did not ameliorate motor symptoms or prevent neuronal loss (30). Nevertheless, intervention to modulate microglial activation states and promote an anti-inflammatory environment may have therapeutic potential in MSA.

To minimize any influence of MSA subtype, we used mixed MSA cases for the remainder of the study in which we investigated cytokine and chemokine protein expression, the CX3CL1/CX3CR1 microglial regulatory pathway and inflammatory gene expression. Tissue availability for such studies precluded the use of small brain regions and, as the frontal lobe is moderately affected in MSA, it was chosen for this part of the study. We were also able to include the cerebellum for gene expression analysis. Previous studies have successfully used postmortem human brain tissue to demonstrate alteration of cytokine expression in various diseases (31–33). Our cytokine array data did not show any significant change in the expression of cytokines and chemokines in MSA. These results are in accordance with our multi-immunofluorescence data which has shown no significant increase in activated microglia in MSA. When examining and interpreting cytokine and chemokine expression we were conscious of several key factors that affect the stability of cytokines measured in postmortem tissue (34). We selected cases that had the shortest possible postmortem delay in order to minimize the degree of cytokine degradation by proteases during this time; however, length of time in −80°C storage may also contribute to their degradation. In addition, we had sought to exclude cases that had concomitant pathologies that might exacerbate any inflammatory response. Both cases and controls may have been exposed to over-the-counter anti-inflammatory medication which may not have been recorded in medical notes and could have effected cytokine and chemokine expression. The cytokine/chemokine response is a fluctuating and rapid process which is quickly modulated by regulatory signaling. These factors may have influenced the study findings.

CX3CL1 or Fractalkine is a novel type of chemokine with a unique CX3C motif (35). CX3CL1 differs from other chemokines as the molecule exists as a membrane-bound glycoprotein with the chemokine attached to the membrane via a mucin-like stalk. CX3CL1 was found to bind with high affinity to a microglial orphan chemokine receptor that was subsequently renamed CX3CR1 (36). CX3CL1 has 2 mechanisms by which it can bind to CX3CR1, either while attached to the cell membrane (100 kDa) or after detaching from the mucin-like stalk to provide a soluble form (60 kDa). Binding of CX3CL1 to CX3CR1 triggers PI3K-dependent Ca2+ influx and activates MAPK and Akt pathways, the binding of membrane bound CX3CL1 has been shown to produce more potent effects than those of soluble CX3CL1 (37). Several reports have shown that CX3CL1 has an inhibitory effect on microglial activation (38–42). Loss of this dampening down of microglial activation via the CX3CL1/CX3CR1 interaction may account for the increase in neurotoxicity in mouse models of both Alzheimer disease, Parkinson disease, prion disease, and amyotrophic lateral sclerosis, which lack CX3CR1 (23, 39, 43–45). Our findings have shown a significant decrease in the soluble 60 kDa form of CX3CL1 in MSA, the form of the molecule with less potent anti-inflammatory properties. This altered expression may reflect modulation of microglial response via alteration of the CX3CL1/CX3CR1 axis in MSA leading to limited microglial activation and proliferation in the surrounding region (23, 46). ADAM10 is required for the cleavage of CX3CL1 and it is released as a soluble molecule (47). This lower level of soluble CX3CR1 expression compared with control may indicate a failure of ADAM10 to permit the release of the molecule in MSA brain (48). The slighter high level of the full-length, membrane-bound 100 kDa CX3CL1 molecule in MSA may indicate this reduced membrane release, however, this increase did not reach significance. Further indication that modulation of microglial activation by CX3CL1/CX3CR1 in MSA comes from our demonstration of increased expression of CX3CR1 in MSA. In the context of data from a model of glaucoma in which an anti-inflammatory agent, crocin, successfully reduced microglial activation via CX3CR1 upregulation, this might indicate an anti-inflammatory effect in MSA (49). These results support our findings that there is no significant alteration in Iba-1 protein in the frontal lobe and inflammatory cytokines were unchanged.

To gain further insight into the inflammatory response in MSA, we analyzed the mRNA expression of a panel of genes related to inflammation. The genes showing a fold change >2 were *ARG1, MASP1, NOX4*, and *PTGDR2* in the frontal lobe and *C6* in the cerebellar white matter. Of these, *ARG1*, with an anti-inflammatory function and *PTGDR2*, with a proinflammatory function, were downregulated. *MASP1*, *NOX4*, and *C6* were upregulated and all have a proinflammatory influence.

Arginase-1 (*ARG1*) mRNA was significantly downregulated in MSA, this correlated with our finding that Arginase-1-positive microglia form a minority of the activated microglial population. Also, downregulated in MSA was Prostaglandin D2 receptor 2 (*PTGDR2/CRTH2*), a G-protein coupled receptor that, when activated, causes chemotaxis of eosinophils, basophils, and T helper type cells in the periphery. Little is understood about the role of PTGDR2 in brain; however, agonist-mediated activation of the receptor was found to exacerbate glutamate neurotoxicity (50). Of the genes that we determined to be upregulated in MSA, the greatest difference compared with control was in the expression of Complement component 6 (*C6*) in the cerebellar white matter; this was the only gene that was found to be significantly altered in the cerebellar white matter. C6 joins a complex with other complement components; this complex forms a pore on the cell surface and allows the influx of Ca^2+^, Na^+^, small molecules, and water, which results in cell lysis (51). It is currently unclear as to why the cerebellar white matter, which is no less affected by the disease than the other areas examined, should show less alteration in the expression of inflammation-linked genes as well as altered expression of a gene which is unaffected in frontal lobe. The cerebellum in MSA may have a different mechanism of response to disease and may, as has been suggested, employ a specialized melatonin-mediated response to inflammation that reduces the inflammatory response (52). Of the genes found to be upregulated in the frontal lobe, Mannan-binding lectin serine protease 1 (*MASP1*) showed the highest fold change. MASP1 acts via protease-activated receptor signaling to cause recruitment of leukocytes (53). Also upregulated in the MSA frontal lobe was Nicotinamide adenine dinucleotide phosphate oxidase 4 (*NOX4*), which has been shown to be upregulated in neurons and endothelial cells after stroke, and its inhibition has been shown to be neuroprotective (54, 55).

In conclusion, our study has shown greater numbers of microglia in the substantia nigra of OPCA cases with no significant difference in number or degree of activation in other brain regions or MSA disease subtypes. Consistent with this data, we see no significant change in cytokines, one of the functional outputs of activated microglia. However, analysis of mRNA shows a significant difference in the expression of a subset of inflammation associated genes, suggesting that some level of inflammatory response is initiated in MSA. Here, we indicate a possible neuronal-mediated regulatory control on the activation and proliferation of microglia. We have shown reduction in the level of CX3CL1 soluble protein and an increase in the microglial receptor of the glycosylated form CX3CR1. These data implicate altered neuronal feedback on microglial activation in MSA, while the increase in CX3CR1 expression may represent a persistent, potentially protective (56) effort to maintain monocyte survival.

Neuroinflammation has many times been described as a double-edged sword: too much leads to catastrophic damage, while too little permits the accumulation of misfolded protein debris and fails to repair injured tissue. Here, we have highlighted alteration in transcription of genes involved in inflammation and we have provided evidence that the neuronal control of microglial activation may be altered in MSA. These findings support the hypothesis that inflammatory mechanisms may be targets for therapeutic intervention in MSA.

## Supplementary Material

Supplementary Data
