# Supplementary figures and images for: Immunohistochemical and Molecular Investigations Show Alteration in the Inflammatory Profile of Multiple System Atrophy Brain
Source: J Neuropathol Exp Neurol. 2018 Apr 23;77(7):598–607. doi: 10.1093/jnen/nly035 (PMC6005028; doi:10.1093/jnen/nly035)

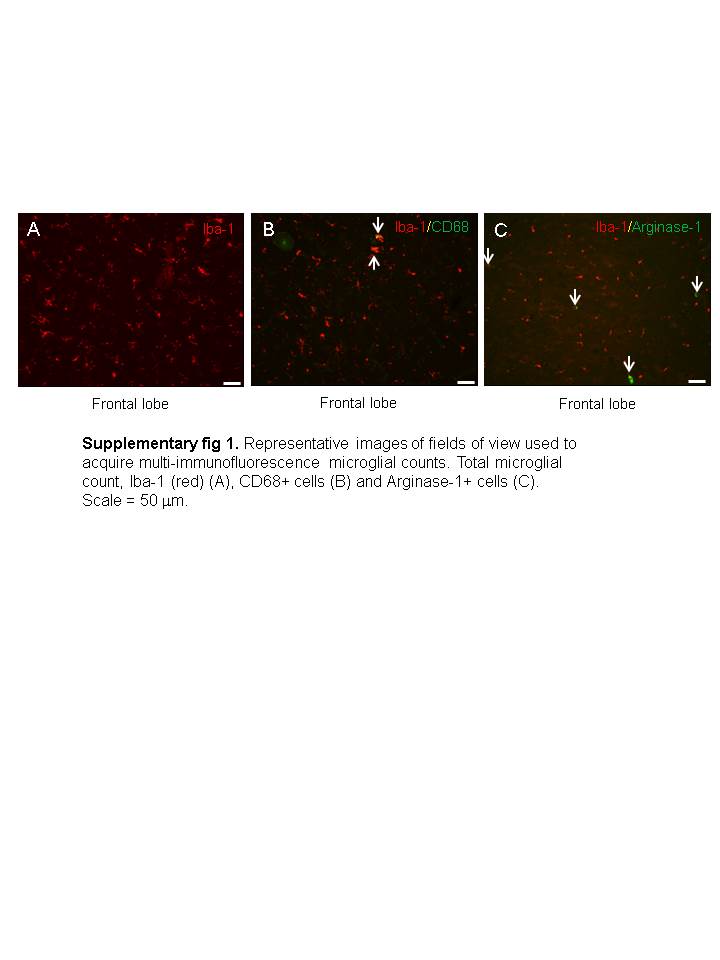

Supplement: Supplementary Data [file nly035_supp.zip › Supplementary figure 1.jpg]

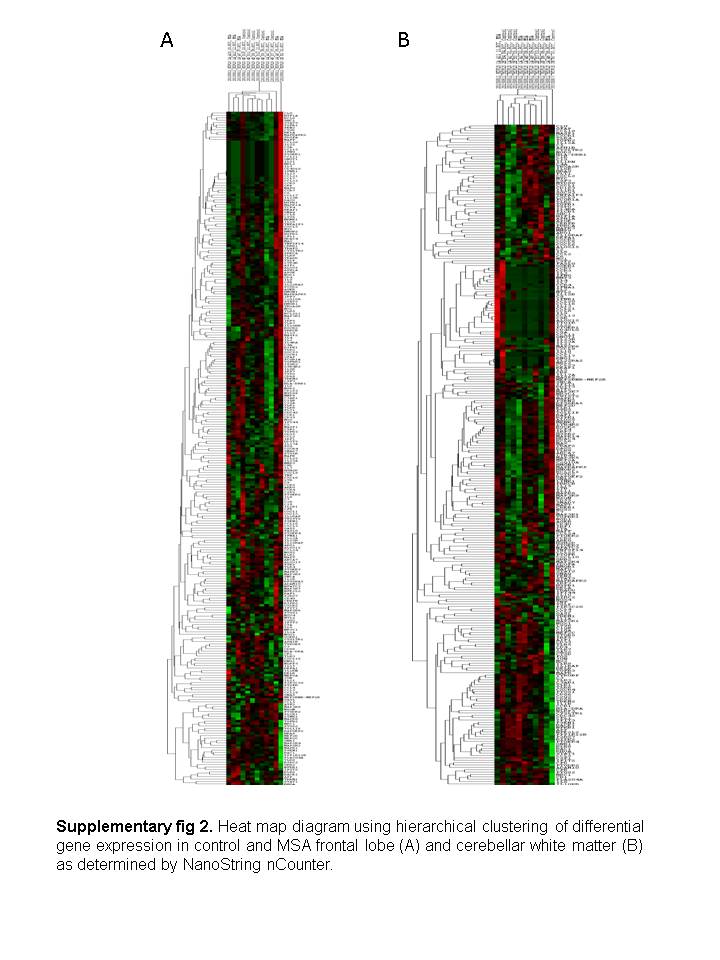

Supplement: Supplementary Data [file nly035_supp.zip › Supplementary figure 2.jpg]
