# Supplementary material for: Immunohistochemical and Molecular Investigations Show Alteration in the Inflammatory Profile of Multiple System Atrophy Brain
Source: J Neuropathol Exp Neurol. 2018 Apr 23;77(7):598–607. doi: 10.1093/jnen/nly035 (PMC6005028; doi:10.1093/jnen/nly035)
Supplement: Supplementary Data [file nly035_supp.zip › Supplementary Table 1.doc]

**Supplementary Table 1. Patient Details for Cases Used in Double Immunofluorescence S**tudy

| **Diagnosis** | **Age (years)** | **Gender** | **PMD (h:min)** |
| --- | --- | --- | --- |
| Control 1 | 79 | F | 240:00 |
| Control 2 | 68 | F | 69:05 |
| Control 3 | 68 | F | 78:15 |
| Control 4 | 57 | M | 15:30 |
| Control 5 | 73 | F | 24:00 |
| MSA SND 1 | 67 | M | 49:40 |
| MSA SND 2 | 70 | M | 62:50 |
| MSA SND 3 | 70 | M | 87:46 |
| MSA SND 4 | 67 | F | 80:05 |
| MSA SND 5 | 68 | F | 50:10 |
| MSA OPCA 1 | 61 | M | 24:00 |
| MSA OPCA 2 | 68 | M | 99:30 |
| MSA OPCA 3 | 66 | F | 75:20 |
| MSA OPCA 4 | 64 | F | 15:30 |
| MSA OPCA 5 | 51 | M | 125:25 |
