# Supplementary material for: Immunohistochemical and Molecular Investigations Show Alteration in the Inflammatory Profile of Multiple System Atrophy Brain
Source: J Neuropathol Exp Neurol. 2018 Apr 23;77(7):598–607. doi: 10.1093/jnen/nly035 (PMC6005028; doi:10.1093/jnen/nly035)
Supplement: Supplementary Data [file nly035_supp.zip › Supplementary Table 2.doc]

**Supplementary Table 2. Patient Details of Cases Used in NanoString (NS), Cytokine Array (CA) and Western Immunoblotting (WB) Studies**

| **Diagnosis** | **Age** | **Gender** | **PMD** | **Technique** |
| --- | --- | --- | --- | --- |
| Control 6 | 80 | F | 51:10 | NS, CA, WB |
| Control 7 | 93 | F | 43:07 | NS, CA, WB |
| Control 8 | 87 | M | 38:50 | NS, CA |
| Control 9 | 88 | M | 16:25 | NS, CA |
| Control 10 | 81 | M | 52:05 | NS |
| Control 11 | 73 | F | 26:00 | NS |
| Control 12 | 38 | M | 82:35 | CA |
| Control 13 | 89 | F | 38:29 | CA |
| Control 14 | 82 | F | 99:20 | WB |
| Control 15 | 93 | F | 89:25 | WB |
| Control 16 | 79 | F | 89:50 | WB |
| Control 17 | 86 | F | 120:45 | WB |
| Control 18 | 69 | M | 168:00 | WB |
| Mixed MSA 1 | 66 | F | 23:20 | NS, CA, WB |
| Mixed MSA 2 | 50 | M | 30:15 | NS, CA |
| Mixed MSA 3 | 63 | M | 20:40 | NS, CA |
| Mixed MSA 4 | 60 | F | 35:30 | NS, CA |
| Mixed MSA 5 | 70 | M | 32:30 | NS |
| Mixed MSA 6 | 65 | F | 34:25 | NS |
| Mixed MSA 7 | 85 | F | 89:00 | CA |
| Mixed MSA 8 | 67 | F | 108:00 | CA |
| Mixed MSA 9 | 68 | F | 36:35 | WB |
| Mixed MSA 10 | 63 | M | 20:40 | WB |
| Mixed MSA 11 | 59 | F | 48:35 | WB |
| Mixed MSA 12 | 69 | M | 53;15 | WB |
| Mixed MSA 13 | 70 | M | 32:30 | WB |
| Mixed MSA 14 | 68 | F | 107:00 | WB |
