# Supplementary material for: Immunohistochemical and Molecular Investigations Show Alteration in the Inflammatory Profile of Multiple System Atrophy Brain
Source: J Neuropathol Exp Neurol. 2018 Apr 23;77(7):598–607. doi: 10.1093/jnen/nly035 (PMC6005028; doi:10.1093/jnen/nly035)
Supplement: Supplementary Data [file nly035_supp.zip › Supplementary Table 3.doc]

**Supplementary Table 3. List of Genes Identified as Having Significantly Altered Expression in MSA Compared to C**ontrol

| Gene name | Upregulation in MSA | Downregulation in MSA | Significance (p value) | Fold Change | Protein Encoded and Function |
| --- | --- | --- | --- | --- | --- |
| Frontal lobe | | | | | |
| *ABCA7* | - | Y | 0.0144 | 1.96 | ATP-binding cassette transporter A7: unknown, possibly lipid homeostasis |
| *ARG1* | - | Y | 0.0215 | 3.64 | Arginase-1: Anti-inflammatory |
| *DDIT3/CHOP* | Y | - | 0.0379 | 1.68 | DNA-damage inducible transcript 3: Induces cells cycle arrest in response to endoplasmic reticulum stress and DNA damage |
| *IL15* | Y | - | 0.0443 | 2.16 | Interleukin 15: Natural killer cell development and function and CD8+ T-cell homeostasis |
| *IL1A* | Y | - | 0.0255 | 2.75 | Interleukin 1A: Induces apopdtosis and response to cell injury |
| *MASP1* | Y | - | 0.0437 | 3.64 | Mannan-binding lectin serine protease 1; Pro-inflammatory |
| *NOX4* | Y | - | 0.0472 | 3.28 | NADPH oxidase 4: Pro-inflammatory |
| *PTGDR2* | - | Y | 0.0186 | 3.10 | Prostaglandin D2 Receptor 2: Pro-inflammatory |
| *SMAD7* | - | Y | 0.0276 | 1.36 | SMAD family member 7: Negative regulator of transforming growth factor β (TGF-β) |
| Cerebellar white matter | | | | | |
| *C6* | Y | - | 0.0436 | 2.88 | Complement component 6: Pro-inflammatory |
